# An exploration of the relationship between recruitment communication and foraging in stingless bees

**DOI:** 10.1093/cz/zoab043

**Published:** 2021-05-12

**Authors:** Robbie I’Anson Price, Francisca Segers, Amelia Berger, Fabio S Nascimento, Christoph Grüter

**Affiliations:** 1 Department of Ecology and Evolution, University of Lausanne, Lausanne 1015, Switzerland; 2 Swiss Centre for Affective Sciences, University of Geneva, Genève 1201, Switzerland; 3 Department for Applied Bioinformatics, Institute of Cell Biology and Neuroscience, Goethe University, Frankfurt 60438, Germany; 4 Departamento de Biologia, Faculdade de Filosofia, Ciências e Letras de Ribeirão Preto, Universidade de São Paulo, Ribeirão Preto, São Paulo CEP 14040-901, Brazil; 5 School of Biological Sciences, University of Bristol, 24 Tyndall Avenue, Bristol BS8 1TQ, UK

**Keywords:** communication, foraging, social information, stingless bee

## Abstract

Social information is widely used in the animal kingdom and can be highly adaptive. In social insects, foragers can use social information to find food, avoid danger, or choose a new nest site. Copying others allows individuals to obtain information without having to sample the environment. When foragers communicate information they will often only advertise high-quality food sources, thereby filtering out less adaptive information. Stingless bees, a large pantropical group of highly eusocial bees, face intense inter- and intra-specific competition for limited resources, yet display disparate foraging strategies. Within the same environment there are species that communicate the location of food resources to nest-mates and species that do not. Our current understanding of why some species communicate foraging sites while others do not is limited. Studying freely foraging colonies of several co-existing stingless bee species in Brazil, we investigated if recruitment to specific food locations is linked to 1) the sugar content of forage, 2) the duration of foraging trips, and 3) the variation in activity of a colony from 1 day to another and the variation in activity in a species over a day. We found that, contrary to our expectations, species with recruitment communication did not return with higher quality forage than species that do not recruit nestmates. Furthermore, foragers from recruiting species did not have shorter foraging trip durations than those from weakly recruiting species. Given the intense inter- and intraspecific competition for resources in these environments, it may be that recruiting species favor food resources that can be monopolized by the colony rather than food sources that offer high-quality rewards.

Foraging in social insect colonies often involves many individuals collecting different types and qualities of food from many different locations. To help with efficient foraging, insect societies have evolved communication systems that allow successful foragers to transfer information about the foraging environment to nest-mates. Impressive examples include the waggle dance in honeybees (von Frisch 1967); tandem running in ants (Franks and Richardson 2006); and pheromone trail recruitment in some ants, termites, and stingless bees (Czaczkes et al. 2015). The use of socially acquired information is thought to be a highly successful strategy because individuals will filter out low payoff behaviors by only demonstrating high payoff behaviors (Grüter et al. 2010; Rendell et al. 2010; Grüter and Leadbeater 2014). For example, foragers of many social insects will modulate communication intensity according to food source profitability (honeybees: von Frisch 1967; ants: Beckers et al. 1993; Jackson and Chaline 2007; Czaczkes et al. 2015; stingless bees: Nieh et al. 2003; Jarau and Hrncir 2009). This means that recruited individuals using this filtered information will have a higher chance of finding a better resource. Indeed, honeybee recruits have been shown to find higher quality resources than scouts who do not use social information (Seeley 1983; Seeley and Visscher 1988). From a colony perspective, it means that more foragers will exploit high-quality resources than low-quality resources.

If species that communicate foodlocations are foraging at the highest quality resources, we might expect that species that do not recruit to a specific resource, and forage solitarily, will on average forage at lower quality resources if they follow a trial-and-error strategy. However, if there are significant benefits to communicating specific food sources (either rewarding flower species or the location of rewarding patches) in terms of exploiting high-quality resources, it begs the question; why do not all species communicate rewarding food sources? While acquiring information socially can be highly adaptive, there are costs in doing so. Social information is not always reliable (Feldman et al. 1996), there is the possibility that it is out of date or inaccurate and noise is universal in information transfer (e.g., Giraldeau et al. 2002; Grüter and Leadbeater 2014). Furthermore, providing signals outside of the nest about food location could attract aggressive bees to the food site (Nieh et al. 2004; Lichtenberg et al. 2014). Another problem might be that individuals may spend longer waiting for social information and therefore costs of using social information would be higher (Seeley 1983; Seeley and Visscher 1988; Dechaume-Moncharmont et al. 2005). Indeed, even in honeybee colonies, the cost of waiting for information may outweigh the benefits that it infers in certain environments (I’Anson Price et al. 2019). As a result, the payoff from using social information is highly variable depending on the environment and could be relatively low in an environment where good food sources are easy to find through individual search (e.g., Dornhaus and Chittka 2004; Seppänen et al. 2007; Beekman and Lew 2008; I’Anson Price et al. 2019).

Stingless bees (Meliponini) comprise more than 550 species (Rasmussen and Cameron 2010; Grüter 2020), all of which are highly eusocial. The density at which they can be found in an environment creates intense inter- and intra-specific competition for limited resources (Johnson and Hubbell 1974; Hubbell and Johnson 1978; Hrncir and Maia-Silva 2013a, 2013b). Stingless bees forage for a number of resources including water, pollen, nectar, plant sap, and resin (Roubik 1989; Grüter 2020). Nectar is the primary carbohydrate source for most stingless bees. It is known that stingless bees show a preference for nectars that contain a higher concentration of sugar (e.g., Biesmeijer and Ermers 1999; León et al. 2015; Peng et al. 2019; Silva et al. 2019). Pollen is the main protein source for stingless bee colonies; however, pollen also contains amino acids, water, and vitamins among its other constituents (Solberg and Remedios 1980).

When foraging, the most significant challenges for stingless bees are potentially competition and a temporally and spatially restricted resource distribution (Johnson and Hubbell 1974; Hubbell and Johnson 1978; Nieh 2004; Hrncir and Maia-Silva 2013a, 2013b). If a species’ recruitment strategy allows a colony to direct foragers to a specific resource, it may be able to monopolize it through high forager number. Mass-recruiting stingless bees are able to recruit using field-based communication to a specific food location. Field-based communication mechanisms include scent trailing, food site marking and, potentially, aerial odor trails reviewed in Lindauer and Kerr (1960); Nieh (2004); Barth et al. (2008); Jarau and Hrncir (2009); and Grüter (2020). In some cases, it is still unknown how foragers are able to recruit nestmates to specific locations (e.g., *Partamona orizabaensis*, Flaig et al. 2016). Mass-recruiting colonies may have bursts of activity as they work to quickly mobilize foragers toward a new resource (Roubik 1989). These species can monopolize a resource by keeping other species or colonies away, either through aggression or through the sheer number of foragers (Hubbell and Johnson 1978; Hrncir and Maia-Silva 2013a, 2013b). Many other species may be able to communicate the presence and/or scent of a food source upon returning to the nest by producing sounds and/or excitatory movements but no information about food location is provided (Kerr et al. 1963; Jarau and Hrncir 2009; Grüter 2020). Some species, like *Plebeia droryana*, show a limited ability to direct nestmates toward the general direction of a food source, but the number of recruited bees remains low (Lindauer and Kerr 1960; Peng et al. 2021). We have termed this group of non-location specific foragers, “weakly recruiting species.” It should be noted that the distinction between mass-recruiting and weakly recruiting species is graded, rather than absolute.

Currently, our understanding of the realized benefits of location-specific mass-recruitment over a more solitary foraging strategy in stingless bees is very limited. We sought to address this by asking 1) if mass-recruiting species collect forage with on average higher sugar content and 2) if the use of social information enables mass-recruiting species to spend on average less time on a foraging trip. Furthermore, we ask if mass-recruitment means that foraging activity is more variable for these species due to bursts of recruitment (Roubik 1989). In this study, we broadly define species as being able to recruit to food locations if they lay pheromone trails (mass-recruiting). We compare these species to species without known trail laying behavior (weakly recruiting). We use these 2 broad categories because we currently lack more detailed information about the foraging methods for most stingless bee species, which would allow for a more precise classification system.

## Materials and Methods

### Study area

The study was performed on the Universidade de São Paulo (USP) campus in Ribeirão Preto (21°10′30 S and 47°48′38 W), Brazil. The University campus is a former coffee farm but retains areas with plants that are native. The campus has a 75-ha forest area planted with species that are typical of the original vegetation (Pais and Varanda 2010). The local climate has 2 well-defined seasons: a cool/dry season starting in May and continuing until September and a hot/wet season from October to April. Data collection was carried out during the hot and wet months of October and November in 2013 (question 1) and February and March in 2016 (questions 2 and 3).

### Bee species

More than 20 species living both wild and in wooden hive boxes can be found on campus. Each question that we asked used a different selection of species depending on the question and accessibility of colonies. All studied colonies foraged on natural food sources and were situated within 1 km of each other. Colony foraging activity was variable between species and reflected the differences in colony size. For further information on the species used see Table 1.

**Table 1.** All species used in the study

Species	Foraging communication	Foraging system	Average foragers leaving/min	Experiment
*Friesella schrottkyi* (Friese, 1900)	No recruitment reported, small colony size, and timid nature	W-R	1.49	Q3
*Frieseomelitta varia* (Lepeletier, 1836)	Intranidal sounds and agitations (Jarau et al. 2003; Lichtenberg et al. 2010)	W-R	6.59	Q1, Q2, Q3
*Nannotrigona testaceicornis* (Lepeletier, 1836)	Localized unspecific chemical cues (Schmidt et al. 2005)	W-R	7.81	Q1, Q3
*Plebeia droryana* (Friese, 1900)	Intranidal sounds, possible weak directional recruitment (Peng et al. 2021)	W-R	6.25	Q3
*Tetragonisca angustula* (Latreille, 1811)	Food source marking and intranidal sounds and agitations (Villa and Weiss 1990; Slaa et al. 2003)	W-R	18.59	Q1, Q2, Q3
*Tetragona elongata*^a^ (Lepeletier and Serville, 1828)	Possible scent marking at feeder (Jarau et al. 2003)	W-R	45.17	Q1, Q3
*Partamona helleri* (Friese, 1900)	Hypothesized partial scent trail marking (Contrera and Nieh 2007)	M-R	8.01	Q3
*Scaptotrigona bipunctata* (Lepeletier, 1836)	Scent trails (Kerr et al. 1963)	M-R	30.64	Q1, Q2, Q3
*Scaptotrigona depilis* (Moure, 1942)	Scent trails (Schmidt et al. 2003)	M-R	26.49	Q1, Q2, Q3
*Trigona braueri* (Friese, 1900)	Most likely scent trail marking (Grüter 2020)	M-R	76.77	Q3
*Trigona hyalinata* (Lepeletier, 1836)	Partial scent trail marking (Nieh et al. 2003)	M-R	50.98	Q3
*Trigona hypogea* (Silvestri, 1902)	Scent trail marking for carcass food sites (Noll 1997)	M-R	n/a	Q1
*Trigona recursa* (Smith, 1863)	Scent trail marking (Jarau et al. 2004)	M-R	14.44	Q1, Q3

Species shaded gray are considered to be weakly recruiting (W-R), unshaded species are considered to be mass-recruiting (M-R). The question column denotes which question(s) a species was used to answer. Foraging activity is the average of foraging counts for 2–5 colonies per species (see Q3). ^a^We note that *T. elongata* corresponds to the form often called *Tetragona clavipes* in studies performed in South-Eastern Brazil (Pedro, 2014).

### Q1. Forage quality

We collected data from 4 species (total of 15 colonies, 2–5 colonies per species) that are known to use mass-recruitment when foraging (*Scaptotrigona depilis*, *Scaptotrigona* *bipunctata*, *Trigona recursa*, and *Trigona* *hypogea*) and 4 species (total of 20 colonies, 5 per species) that are weakly recruiting (*Nannotrigona testaceicornis*, *Tetragonisca angustula*, *Frieseomelitta varia*, and *Tetragona elongata*). We collected 37 ± 10.3 (mean ± SD) returning foragers per species between 10 am and 2 pm. After collection, bees were cooled and immobilized slowly (2–5 min) to reduce the number of bees regurgitating their load. This was done by placing them inside a polystyrene box inside a freezer at −20°C. Liquid food (which could be nectar, honeydew, fruit juice, or water) was extracted by applying gentle pressure to the abdomen of the bee and holding a capillary tube to its mouth. The weight of a bee was taken 3 times: 1) collected state, 2) after removal of resin and/or pollen, and 3) after the extraction of liquid from the bee (Sartorius TE64 high precision balance). The extracted liquid was then placed on a refractometer (Kern-ORA80BE) and the sugar content percentage was taken from each, full load. Between readings the refractometer was cleaned thoroughly with water then dried. The refractometer measures the percentage in weight of sugar per unit volume. Measurement errors can occur due to the non-sugar constituents of the liquid and variability in the environment in which the reading was taken (Inouye et al. 1980; Roubik 1989). We did our best to control the temperature and humidity while measuring the sugar concentration of liquid loads from the bees by performing the measurements in the laboratory on days with similar weather conditions. Corbet (2003) found that depending on whether the liquid is fructose, sucrose, or glucose, the error in sugar content is about 3–4% (Corbet 2003). Information on the pollen, nectar, and resin quantity was not recorded due to the low quantities collected by the often-small bees.

### Q2. Forage trip duration (collection period 2)

Following the results of collection period 1 (mass-recruiting species, in particular the 2 *Scaptotrigona* species, did not return with higher quality forage than weakly recruiting species), we tested the hypothesis that these mass-recruiting species might instead favor nearby food sites that they can quickly exploit. We chose 4 stingless bee species for this experiment: the 2 trail-laying species that returned with the lowest average sugar content in their forage (*S.* *bipunctata* and *S. depilis*) and 2 weakly recruiting species that returned with forage significantly higher in sugar content than these trail-laying species (*F.* *varia* and *T.* *angustula*). We studied 3 colonies per species. For each colony around 30 foragers were given unique markings on their thorax using acrylic paint. To record the time it takes for a bee to leave the colony and return, colonies were filmed for 90 min in the morning (from around 10 am) and 90 min in the afternoon (from around 2 pm) the day after marking. Any trip durations lower than 90 s were not considered to be nectar or pollen foraging trips (potentially orientation flights) and were removed from the analysis. For an individual bee, foraging journey times on a day were averaged when carrying out analyses. Data were collected from 54.75 ± 8.2 foragers per species.

### Q3. Diurnal foraging activity (collection period 2)

Foraging activity was collected from 12 species over the course of the day on 2 days with normal foraging conditions for this time period (4 March 2016 and 8 March 2016). Recruitment intensity was expected to change considerably from day to day in mass-recruiters (e.g., honeybees, Seeley 1995 ), whereas weakly recruiting species were expected to have a more consistent foraging activity during these 2 days. For each species, we collected data from 3 colonies (with the exception of using 2 colonies of *Trigona elongata* and *Trigona* *hyalinata*). Each colony entrance was filmed for 1 min every hour to record the number of outgoing bees. Data collection started just after sunrise (around 06:15) and finished just after sunset (around 18:45). These data were used to assess relative colony size (see Table 1) and test the influence of colony size on variation in foraging activity. The videos were observed using VLC player (v2.2.6). Count data at each time point were converted into foraging activity percentages for the day (foraging activity for all time points on a day sum to 100%).

### Statistical analyses

We used R 3.1.0 (TeamR 2011), “MCMCglmm” (Hadfield 2010), “lme4” (Bolker et al. 2009), and “lmerTest” to perform general and generalized linear mixed-effect models (LMEs and GLMMs) on the liquid sugar concentration [Q1] and foraging trip duration [Q2]. For [Q1], when using foraging system (mass-recruiting versus weakly recruiting) as a fixed effect, Markov chain Monte Carlo (MCMC) methods were used. This allowed us to use the phylogenetic relationships, that is, evolutionary closeness, for the random effects structure. We used the stingless bee phylogeny from Rasmussen and Cameron (2010) for all phylogenetic controls (Rasmussen and Cameron 2010). Colony was also used as a random effect, taking into account that data for a species were taken from more than 1 colony. We ran 49,501 iterations, using a thinning interval of 500 and a sample size of 100. Foraging trip duration [Q2] times for an individual bee were averaged when carrying out statistical analyses. For the model used in [Q2], we used MCMC methods, again allowing us to use phylogeny as a random effect as well as colony. Recruitment system was used as the fixed effect. We ran 49,601 iterations, using a thinning interval of 500 and a sample size of 100. To look at differences between species in both nectar concentration and journey times, we carried out GLMMs using “lmer.” Species was used as the fixed effect in both models. We log transformed the response variable to ensure normality of residuals, this was confirmed using visual inspection. Genus and colony were used as random effects. Genus was used to acknowledge a closer phylogenetic relationship between some species and colony was used because we used several bees from each colony. Pairwise comparisons were carried out between species using the package “multcomp” (Torsten Hothorn 2008) with sequential Bonferroni *P*-value adjustment.

All foraging activity analyses [Q3] were carried out using “lme4” (Bolker et al. 2009) and “lmerTest” to perform GLMMs. First, we tested if there was a difference in variation of colony activity at a time point between days for the 2 foraging systems (mass-recruiting or weakly recruiting). For example, if colony A on day 1 carries out 20% of its total foraging activity at 9 am, how different is this on day 2 for the same colony and time point? The score for a colony was the difference in percentage foraging activity between the 2 days. We log-transformed the response variable to ensure normality of residuals and used foraging system (mass-recruiting or weakly recruiting) and total daily species foraging activity as the fixed effects. Total species foraging activity was used to assess if colony size (which would differ significantly between species) affected foraging activity variation between days. We used species nested within genus as random effects.

We also looked at within species variation at a time point on a data collection day. The question we asked was do colonies within a species show similar activity levels at time points over the day? To do this, we calculated the mean and standard deviation of the percentage foraging activity for a species at each time point on a day and used this to give a coefficient of variation (SD/mean) for each species at a time point. This coefficient of variation at each time point was used to compare between foraging systems. We log-transformed the response variable and once more foraging system and species foraging activity were used as fixed effects. We used genus and day (day 1 or day 2) as random effects in the model.

## Results

### Forage quality

**Figure 1.**
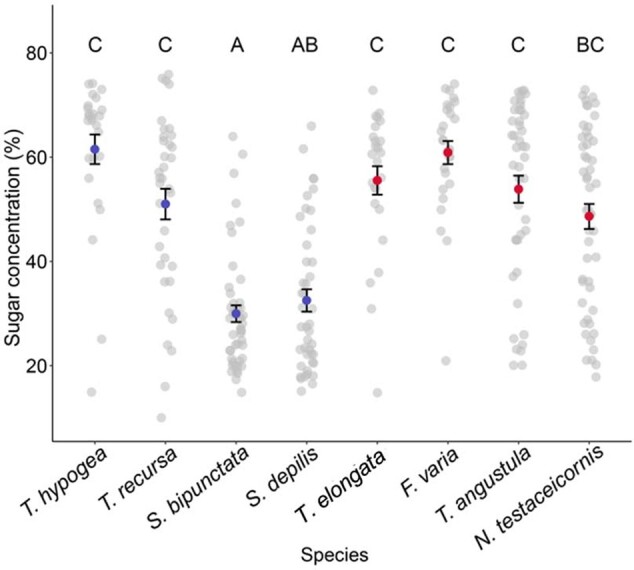
Average sugar concentration of liquid in returning foragers (mean ± *SE*). Blue points represent mass-recruiting species, red points represent weakly recruiting species and gray points represent raw data. Different letters above the data indicate that groups are significantly different (*P < *0.05). Groups with the same letter do not differ.

The average sugar concentration of liquids collected was 40.9 ± 18.7% for mass-recruiting species and 53.6 ± 16.2% for weakly recruiting species. There was no significant difference in sugar content of liquids in returning bees between mass-recruiting and weakly recruiting species (Figure 1) (MCMC *P = *0.36). The 2 mass-recruiting species *S.* *bipunctata* and *S. depilis* returned with liquids that contained the lowest average sugar content (30 ± 11.2% and 32.5 ± 14.1%, respectively). Of the 4 weakly recruiting species, *T.* *elongata*, *F.* *varia*, and *T.* *angustula* all returned with forage of significantly higher sugar content than both of these 2 mass-recruiting species and *N.* *testaceicornis* returned with forage of higher sugar content than *S. bipunctata* (*S. bipunctata* versus: *T. elongata*; *z = *4.084, *P = *0.001, *F. varia*; *z = *5.14, *P < *0.0001, *T. angustula*; *z = *3.935, *P = *0.002, *N. testaceicornis*; *z = *3.226, *P = *0.024, *S. depilis* versus: *T. elongata*; *z = *3.568, *P = *0.008, *F. varia*; *z = *4.613, *P = *0.0001, *T. angustula*; *z = *3.338, *P = *0.014, *N. testaceicornis*; *z = *3.121, *P = *0.062 all *P*-values from pairwise comparisons corrected with sequential Bonferroni). *Trigona hypogea* returned with significantly sweeter liquids than both *S. depilis* and *S. bipunctata* (*S. depilis*; *z = *3.512, *P = *0.009, *S. bipunctata*; *z = *3.936, *P = *0.002). *Trigona recursa* returned with significantly sweeter liquids than *S. bipunctata* (*z* = 3.357, *P = *0.016).

### Foraging trip duration

**Figure 2.**
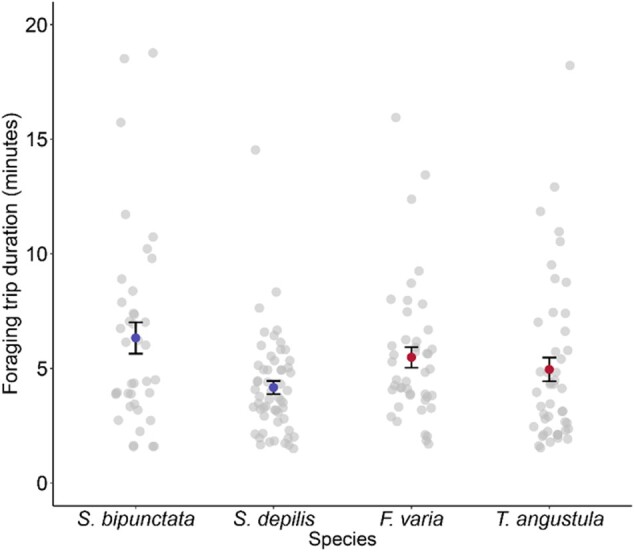
Average foraging trip duration of a bee (mean ± SE). Gray points are mean foraging trips for individual bees, blue points represent mass-recruiting species and red points represent weakly recruiting species.

We did not find a difference in foraging trip duration between foraging systems (MCMC *P = *0.86). Species, also, did not significantly differ in foraging trip duration (Figure 2) (GLMM: *χ*^2^ = 4.05, df = 3, *P = *0.26). *Scaptotrigona depilis* had, on average, the shortest foraging trips (mean ± SD) (4.17 ± 2.15 min). The longest foraging trips were seen in *S. bipunctata* (6.33 ± 4.27 min).

### Foraging activity

**Figure 3.**
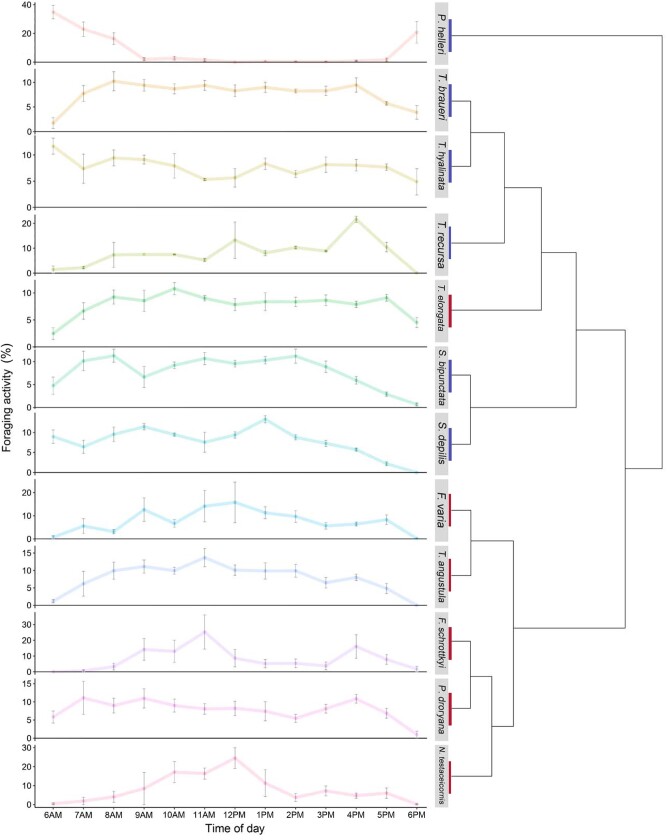
Foraging activity data. Points represent the mean percentage of total daily foraging activity ± SE. Phylogenetic relationships taken from Rasmussen and Cameron (2010) show that the mass-recruiting species are clustered together with the exception of *P. helleri*. The weakly recruiting species are clustered together with the exception of *T. elongata*.

Most species had little activity at the earliest and latest parts of the day (Figure 3). In these species, foraging activity increased over the morning and decreased from around 4 pm. There were some exceptions to this pattern. *Partamona helleri* was only active in the early morning and late afternoon, *T.* *hyalinata* had a fairly constant activity level throughout the day and the activity of *S.* *depilis* was constant from the morning to the early afternoon, then decreased gradually until the evening.

**Figure 4.**
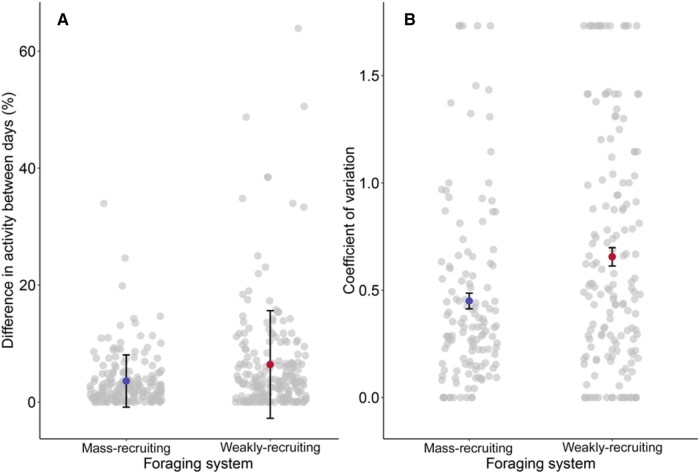
Variation in foraging activity between days. Blue points represent mass-recruiting colonies, red points represent weakly recruiting colonies, and gray points represent raw data. (A) Within colony variation between days, mean ± *SD* (standard deviation used as standard error was too small for the scale). (B) Within species coefficient of variation, mean ± *SE*.

When comparing colonies’ foraging activity at each time point between days, we found that total species foraging activity (proxy for colony size) interacted significantly with foraging system (GLMM: *χ*^2^ = 5.12, *df* = 1, *P = *0.024) (Figure 4A). Larger, mass-recruiting colonies showed a small effect of colony size on foraging variation between days with smaller colonies having greater variation between days. Smaller, weakly recruiting colonies showed a very strong effect of colony size with smaller colonies showing much greater variation. We also looked at within species variation in foraging activity on a day and found that there was no interaction between foraging system and total species foraging activity with regard to their effect on the coefficient of variation (GLMM: *χ*^2^ = 2.44, *df* = 1, *P = *0.12). Total species foraging activity was used to understand if colony size was affecting foraging activity variation over a day. We did not see differences in the coefficient of variation depending on the foraging system (Figure 4B) (*χ*^2^ = 0.93, *df* = 1, *P = *0.34), furthermore, total species foraging activity also did not affect the coefficient of variation (*χ*^2^ = 1.62, *df* = 1, *P = *0.20).

## Discussion

Contrary to our expectation, we found that mass-recruiting species did not return to the colony with higher quality forage than weakly recruiting species. The 2 species that collected the liquid food with the lowest sugar content, *S.* *bipunctata* and *S. depilis* (Figure 1), are known to have strong and precise recruitment to food sources (Kerr et al. 1963; Schmidt et al. 2003). The species that collected the sweetest liquid food was *F.* *varia*, a bee that is not known to recruit nest-mates to a specific food source (Jarau et al. 2003; Lichtenberg et al. 2010). While behavior in social insects is closely tied to foraging energetics and bees should try to maximize energy intake and minimize energetic output (Hrncir and Maia-Silva 2013a), the 2 *Scaptotrigona* species appear to favor something other than high sugar content when foraging. This is somewhat unexpected because *S. depilis* is known to reduce foraging when confronted with reducing energetic returns at experimental feeders (Schmidt et al. 2006). Why did both *Scaptotrigona* species collect liquid food of relatively low energetic value if higher quality food sources are available? Food patch size is potentially an important resource trait for mass-recruiting species as there is reduced benefit in recruiting many foragers to small food patches. *Scaptotrigona* are considered non-aggressive mass-recruiters (Hrncir and Maia-Silva 2013a, 2013b). It may be that creating and maintaining a monopoly on a large resource by recruiting large numbers of bees is more important for non-aggressive species even if the nectar quality is relatively low. Interestingly, both *Scaptotrigona* species used in this study forage at fewer than half the number of flower species at which *T.* *angustula* forages (Ramalho 1990; Wilms et al. 1996; see also Biesmeijer and Slaa 2006). It has been shown that mass-recruiters have a limited capacity of discovering new food sources due to their recruitment to and local enhancement at a food site (Hubbell and Johnson 1978; Biesmeijer and Slaa 2004). This feedback may mean that they continue to exploit a low-quality resource even after a higher quality resource becomes available (Schmidt et al. 2006).

We define quality as the sugar concentration of a liquid, though acknowledge that there are other characteristics of liquid food sources that likely play a role in how a species forages (e.g., imbibing rate, Roubik and Buchmann 1984; distance from the nest, Roubik 1989; León et al. 2015; or the presence of secondary compounds, Singaravelan et al. 2005; Couvillon et al. 2015). It is also the case that certain nectar sources are not accessible to some species, for example, proboscis length will affect the flower species upon which a bee species can forage (Hrncir and Maia-Silva 2013a). Differences in bee morphology may also play a role in the sugar concentration of nectar collected by a species. Harder (1985) reports that corolla depth is positively correlated with nectar sugar concentration allowing some species access to nectar with higher energetic content but at the cost of longer handling time for the foraging bee (Harder 1985). Body size, however, was similar in our species (see Grüter et al. 2017), which makes it unlikely that it explains differences in sugar concentration.

Though we did not collect data on the level of sun exposure on nectar collection days, this may have influenced the nectar quality collected by bees in our sample. The light-colored *Melipona beecheii* from Central America has been shown to collect higher concentration nectar than the dark-colored *Melipona* *costaricensis* due to being able to forage more easily at sunlit patches due to its heat-reflective body color (Biesmeijer et al. 1999). The nectar of higher sugar concentration it collects is a product of water evaporation from the nectar in flowers in these patches. Of our study species, *T.* *elongata*, *F.* *varia*, and *T.* *angustula* are light-bodied. Each returned with liquid food of significantly higher sugar concentration than both dark-bodied *Scaptotrigona* species (Figure 1). However, the species that returned with the highest quality liquid food was the black mass-recruiting *T.* *hypogea* (Figure 1). Little is known about the carbohydrate sources visited by this species. There is no record of it collecting nectar from flowers, therefore, it does not appear to compete for nectar with the other species in this study (Noll 1997). Furthermore, because we are not studying the collected liquid in more detail, we cannot exclude that there may be non-sugar constituents in the liquid collected by *T. hypogea* that affect the refractometer reading (Inouye et al. 1980). For this reason, we are cautious in our evaluation of this data.

Another important factor is the distance to the food source as this will impact the energetic costs of foraging and may also affect how effective recruitment is. Given that we found *Scaptotrigona* species returning with low-quality forage, it may be that some mass-recruiters favor nearby food sources over high-quality food sources. We therefore compared the trip durations of foragers of both *Scaptotrigona* species to the weakly recruiting *T. angustula* and *F. varia*; however, the amount of time spent on a foraging trip did not differ between the 4 species we studied (Figure 2). The average trip duration over all species that we studied was 5 min and 23 s, which is similar to the foraging trip durations of other stingless bees (Wille and Orozco 1975; De Bruijn and Sommeijer 1997, but see Harano et al. 2020 and Maia-Silva et al. 2021 for longer trip durations in *Melipona subnitida*). The short time spent on a trip could suggest that in this highly competitive environment, fast exploitation of a resource is important or, alternatively, foraging “territories” are small due to intense competition. It also further highlights that stingless bees do not usually travel far from the nest when foraging (usually a few hundred meters, Araújo et al. 2004; Grüter 2020) and so their realized niche is highly dependent on the local flora and fauna. Honeybees, on the other hand, can travel more than 10 km when searching for food sources (von Frisch 1967; Beekman and Ratnieks 2000). Their ability to precisely guide nest-mates to a distant food source using waggle dance communication is thought to explain why traveling so far can still be adaptive (Beekman and Ratnieks 2000; Ratnieks and Shackleton 2015). To our knowledge, mass-recruiting stingless bees can only guide nest-mates by scent marking the environment, a strategy that is unlikely to work over long distances.

Contrary to our expectation, there were no differences between the 2 foraging systems in within species variation at time points on a day (Figure 4B). In principle, mass-recruitment allows a colony to quickly mobilize its foragers when a favored food source becomes available and in these moments we might expect a spike in activity (Roubik 1989). However, if mass-recruiting species create a monopoly on food sources, the colony creates a constancy in food source availability that allows them to forage at constant rates for sustained periods. If this is the case we would not see the bursts of activity that have been hypothesized (Roubik 1989). It should be noted that these data were collected on just 2 days and we therefore treat it with caution; a study over several days in a short space of time would offer a better insight into variation in colony foraging activity.

The most unusual foraging activity was observed in *P.* *helleri*, whose activity was high only in the early morning (6 am–8 am) and early evening (6 pm), unlike any other species we studied (Figure 3). Interestingly, Keppner and Jarau (2016) found a similar activity pattern in another *Partamona* species, *P. orizabaensis* (Keppner and Jarau, 2016) . Wilms et al. (1996) report that the trophic niche of *P. helleri* overlaps with *Apis* *mellifera scutellata* more than it does with any of the 11 species of stingless bee used in the study. Of the stingless bees studied, *P. helleri* also had the highest index of competition (Wilms et al. 1996). This may be evidence that this species attempts to reduce competition by foraging in the early and late hours of the day.

Many species-specific differences in foraging-related traits have not been quantified here, which could explain why we did not find foraging patterns that are associated with recruitment communication about food source locations. One potentially important factor that we have not discussed in detail is colony size. Larger colonies tend to recruit nestmates to a food source (see Figure 10.10 in Grüter 2020), and they are able to create and maintain a scent trail and monopolize the resource. For small colonies, recruitment by pheromones would be less effective due to low forager numbers (Beekman et al. 2001). Thus, in future studies, the role of colony size for foraging success deserves more attention. Studies have shown that the tropical environment in which this study was conducted is typically resource-limited and diet overlap is considerable (Hubbell and Johnson 1978; Wilms et al. 1996; Hrncir and Maia-Silva 2013a, 2013b); therefore, it is likely that the intricate dynamics between species (variation in body size, body color, colony size, and communication system of different species coupled with spatial and temporal changes in food source availability) create foraging niches (Hubbell and Johnson 1978; Roubik 1989; Ramalho 1990).

In honeybees, recruitment communication is used for resources of high quality and this creates a filtering mechanism for potential recruits (von Frisch 1967; Seeley 1983). As a result, honeybee foragers that use communicated information often find food of higher quality than foragers that do not rely on communication (but see I’Anson Price et al. 2019). In stingless bees, recruitment to food source location does not seem to lead to significantly better food sources. The mass-recruiting species in our study tended to be more closely related to each other than to weakly recruiting species and Grüter (2020) recently suggested that mass-recruitment by means of pheromone trails may have evolved only once in Neotropical stingless bees, 35–40 million years ago. While we might never know the factors that drove the evolution of trail laying in stingless bee ancestors, future studies can uncover how mass-recruitment can benefit present-day stingless bees.

## Author contributions

R.I.P., F.S., F.S.N., and C.G. contributed toward design, data collection, data analysis, and writing. R.I.P. and A.B. contributed to video analysis.
